# Proportion of At-Risk Alcohol Consumers According to the New French Guidelines: Cross-Sectional Weighted Analyses From the CONSTANCES Cohort

**DOI:** 10.3389/ijph.2024.1606481

**Published:** 2024-02-16

**Authors:** Rita El Haddad, Adeline Renuy, Emmanuel Wiernik, Maria Melchior, Marie Zins, Guillaume Airagnes

**Affiliations:** ^1^ Université de Versailles Saint-Quentin-en-Yvelines, INSERM UMS 11 Cohortes Epidémiologiques en Population, Villejuif, Île-de-France, France; ^2^ INSERM UMS 11 Cohortes Epidémiologiques en Population, Villejuif, Île-de-France, France; ^3^ Université de Sorbonne, INSERM U 1136 Institut Pierre Louis d’Epidémiologie et de Santé Publique, Paris, Île-de-France, France; ^4^ INSERM, Population-based Epidemiological Cohorts Unit, UMS 011, AP-HP.Centre-Université Paris Cité, Paris, France

**Keywords:** prevalence, at-risk alcohol use, sociodemographic factors, clinical factors, French population

## Abstract

**Objective:** To estimate the proportion of the participants of the French national population-based CONSTANCES cohort exceeding the new low-risk drinking guidelines according to sociodemographic and clinical factors.

**Methods:** From 34,470 participants with follow-up data in 2019, among volunteers aged 18–69 years and invited to enroll in the CONSTANCES cohort in 2016 and 2017, weighted prevalence and odds ratios with 95% confidence intervals (CI) exceeding the guidelines using logistic regressions were presented stratified for age, gender, education, occupational grade, employment, income, marital status, pregnancy, work stress, depression, alcohol dependence, binge drinking, cannabis use, smoking status, e-cigarette use, cardiovascular diseases, and cancer.

**Results:** The guidelines were exceeded more by men at 60.2% (95%CI: 59.3%–61.0%) than by women at 36.6% (95%CI: 35.9%–37.4%). Exceeding the guidelines increased with age, socioeconomic status, smoking, vaping, using cannabis, binge drinking, and alcohol dependence. Being depressed was associated with exceeding the guidelines in women. Even though pregnant women were less likely to exceed the guidelines, 7.6% (95%CI: 5.4%–10.6%) were at-risk drinkers.

**Conclusion:** These findings highlight the need to implement effective prevention measures for at-risk alcohol use among the French population.

## Introduction

Alcohol consumption is a major risk factor for premature death and is accountable for more than 200 somatic and psychiatric diseases including cancers, cardiovascular diseases, liver cirrhosis, violence, and suicide [1, 2]. The Global Burden of Disease Study of 2016 demonstrated that there is no level of consumption that minimizes health loss and that the risk of cancers in particular and all-cause mortality in general increases with increasing levels of consumption [3]. Beyond health consequences, high alcohol consumption has as well significant societal and economic consequences at an individual and community level [2]. Moreover, sociocultural factors have an important influence on alcohol consumption and cultural norms vary considerably across society [4].

The highest levels of *per capita* alcohol consumption and the highest proportion of drinkers in the world are observed in the WHO European Region [1]. In France, where 43 million are consumers, alcohol is part of the cultural and social norms and is regularly consumed during social interactions [2]. Alcohol consumption is more common among men than among women [2].

In order to limit health hazards associated with alcohol consumption, many countries like Australia, the United Kingdom, Denmark, and Canada have updated their guidelines and lowered their benchmarks for alcohol consumption that the general population should not exceed over the last decade [5–8]. For instance, Canada’s new low-risk drinking guidelines adopted in 2023 drastically reduce the amount of alcohol consumption considered safe (at most two drinks per week for low-risk drinking for men and women) [5].

In France, in light of the new findings regarding the detrimental role of low to moderate levels of alcohol consumption on health, particularly in view of the increased risk of cancer even at low consumption levels, an expert group commissioned by the French national public health agency (Santé Publique France, SPF) and the French National Cancer Institute (Institut National du Cancer, INCa) proposed new guidelines for alcohol consumption in 2017 [9, 10]. These new guidelines were based on modeling to define the level of alcohol use below which the “absolute lifetime” risk of alcohol-attributable mortality for the French population was between 1% and 1‰, which was considered a tolerable risk [10]. These guidelines consist of a combination of three benchmarks that should not be exceeded to maintain low-risk drinking, as follows: 1/ no more than 10 standard drinks per week, 2/ no more than two standard drinks per day, and 3/ at least two alcohol-free days every week [2, 10]. Estimating the prevalence of the French general population who exceeds these new guidelines while considering a broad range of sociodemographic and clinical factors would be particularly helpful for public health policymakers in refining and monitoring their prevention strategies.

Using 2019 data from the CONSTANCES cohort (Cohorte des consultants des Centres d’examens de santé- Cohort of visitors to health examination centers), a French national longitudinal population-based cohort, we estimated the proportion of the participants who exceed the new guidelines of low-risk drinking in men and women separately. The CONSTANCES cohort includes a sufficient sample size to further stratify the population according to sociodemographic factors (i.e., age, education, income, occupational grade, employment, marital and parental status), alcohol-related factors (history of heavy episodic drinking, the existence of dependence criteria), other substance use (tobacco smoking, vaping, cannabis use), and other health conditions (self-rated health, depression, cardiovascular diseases, and cancers). In addition, we thought to examine separately each of the three criteria of low-risk drinking [11]. Finally, the estimates of exceeding the previous low-risk drinking guidelines were also calculated in order to highlight the impact of the new guidelines on the proportion of people considered to be at-risk drinkers within the general French population [12].

## Methods

### Participants

CONSTANCES is a research infrastructure that aims to facilitate analytical epidemiological work and to enable public health and epidemiological surveillance studies. CONSTANCES consists of a national population-based cohort of randomly recruited participants aged 18–69  years at enrollment. Participants were recruited in 21 selected national health insurance medical screening centers from the principal regions of France [11]. To be recruited, participants must be covered by the general health insurance scheme restricted to salaried workers, employed or retired, and their families, thus excluding agricultural and self-employed workers who are affiliated with other health insurance funds (about 10% of the French population). Between 2012 and 2021, around 220,000 volunteers were included in the CONSTANCES cohort. At baseline and then annually, the participants are invited to complete self-administered questionnaires assessing sociodemographic factors, occupational conditions, and health-related behaviors including alcohol, tobacco, and cannabis consumption [11]. The detailed design and methodology of the CONSTANCES cohort are available elsewhere [13, 14]. CONSTANCES was authorized by the French Data Protection Authority (Commission Nationale de l’Informatique et des Libertés, CNIL) and approved by the Institutional Review Board of the National Institute for Medical Research–INSERM (No. 01–011). All the participants provided an informed consent.

**FIGURE 1:**
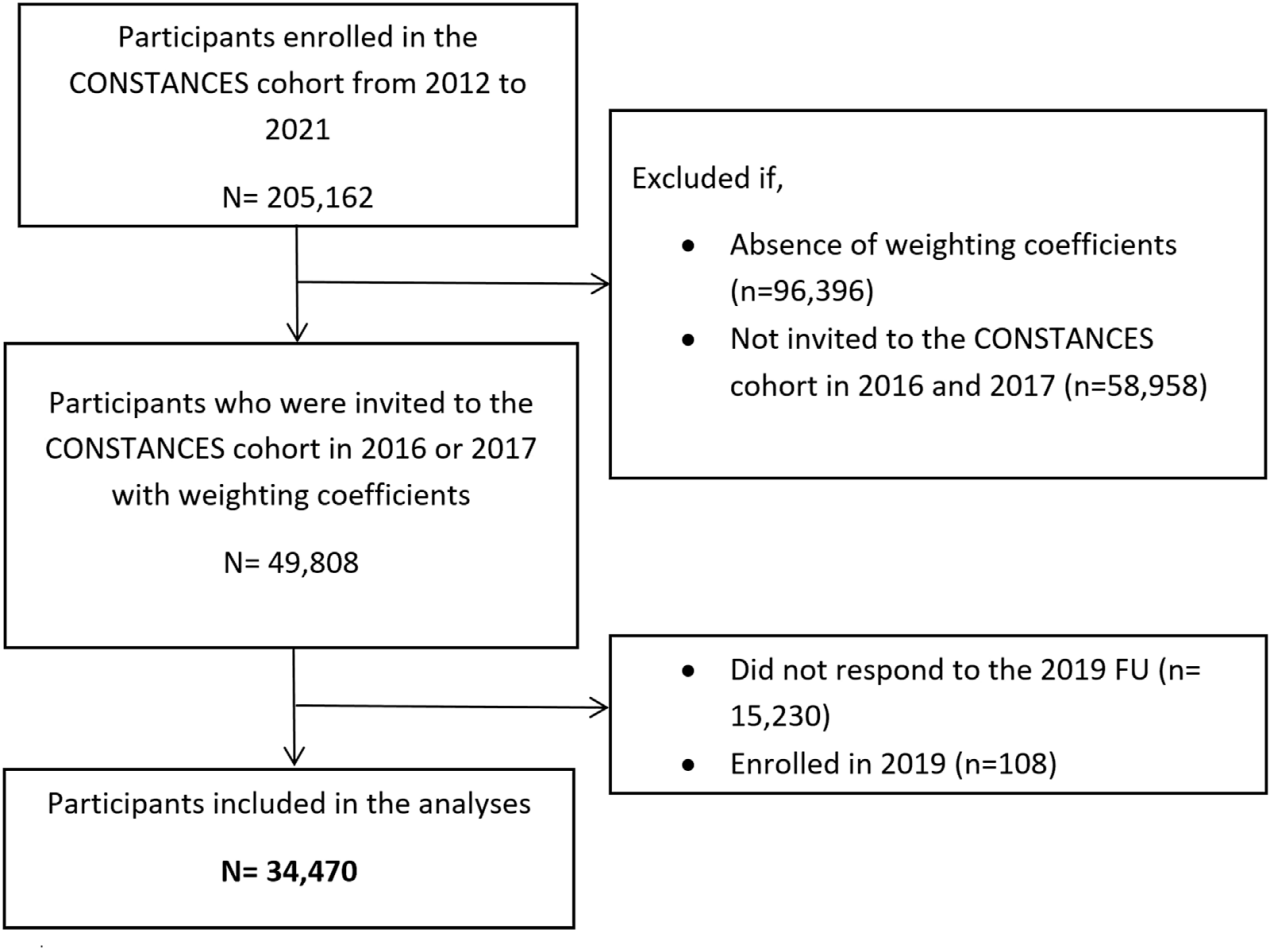
Flow chart of population selection (The CONSTANCES study, Metropolitan France, 2019).

At the time we performed the analyses for the present study, the most recent year for which follow-up data were available was 2019. Thus, in the present study, we selected participants who were invited to join the CONSTANCES cohort in 2016 or 2017 and for whom weighting coefficients were computed (*n* = 49,808). We excluded those who did not respond to the follow-up of 2019 (*n* = 15,230) and those enrolled in 2019 (*n* = 108), however, our analyses considered the probability of non-response during follow-up. Thus, a total of 34,470 participants were included in the present study (Figure 1).

### Measurement of the New French Guidelines For Low-Risk Drinking

Participants are asked if they consumed alcohol over the preceding week and if yes, to report their number of drinks per day for each type of alcoholic beverage (i.e., beer or cider, wine or champagne, spirits, aperitif, premix, and cocktails). They reported their average daily consumption from Monday to Thursday, and their consumption on Friday, on Saturday, and on Sunday. From the latter, the variables for exceeding each criterion were computed as such.

#### Criterion of Exceeding Two Drinks Per Day

Participants who reported consuming more than two drinks per day from Monday to Thursday or on Friday, Saturday, or Sunday were considered as exceeding this criterion.

#### Criterion of Exceeding 10 Drinks Per Week

The total number of drinks per week was computed by adding the average number of drinks per day from Monday to Thursday, multiplied by four, and the number of drinks on Friday, Saturday, and Sunday. If this value exceeded 10, participants were considered as exceeding this criterion.

#### Criterion of Not Having at Least Two Alcohol-Free Days Per Week

From Monday to Thursday, we attributed each reported drink to a day since we did not have data for the number of drinks separately for each day. Thus, participants who reported consuming:

─ Three drinks or more from Monday to Thursday and one drink or more on Friday, Saturday, and Sunday
─ Four drinks or more from Monday to Thursday and one drink or more on at least 2 days from Friday to Sunday

Were considered as exceeding this criterion.

### Sociodemographic Factors

We considered the following sociodemographic variables: age, gender, employment status, education, occupational grade, household income, and marital status.

Participants’ age was divided into five groups (i.e., 20–34, 35–44, 45–54, 55–64, and 65–74 years old). Employment status in 2019 was self-reported and grouped into 1) employed, 2) unemployed, 3) retired, or 4) student. Education at eight levels and grouped into five categories based on the International Standard Classification of Education (ISCED 2011) was used as a self-reported baseline [15]. Occupational grade was also self-reported at baseline and grouped into 1) never worked, 2) manual worker or employee, 3) intermediate profession, and 4) executive. Household income reported at baseline was divided into five categories: 1) less than 2,100, 2) (2,100–2,800), 3) [2,800–4,200] and 4) ≥4,200 euros. In the CONSTANCES cohort, information regarding marital status and children at follow-up was only collected in 2018. Therefore, we considered the latest reported marital status (in a couple or not) and children (yes or no) which was in 2018.

### Clinical Factors

We considered the following clinical characteristics: pregnancy, effort-reward ratio, depression, alcohol dependence, frequency of binge drinking, cannabis use, smoking status, vaping and tobacco use, cardiovascular diseases, and cancer.

Being pregnant and treatment for depression were self-reported in the 2019 follow-up questionnaire. Effort-reward imbalance (ERI) was assessed at baseline to identify stressful psychosocial work environment [16, 17]. Alcohol dependence during the preceding 12 months was measured at baseline using the Alcohol Use Disorder Identification Test (AUDIT) by adding the score of items 4 to 10 and analyzed as tertiles [18, 19]. During the preceding 12 months, frequency of binge drinking [1] never, 2 at least once, self-rated health (from 1 = “Very good” to 8 = “Very Poor” with a cutoff of 3 representing around 80% of the participants with a score ≤3), cannabis use (i.e., not during the previous 12 months, at least once during the previous year), smoking status (i.e., non-smoker or occasional smoker and smoker), and vape and tobacco use (i.e., no use, vape only, tobacco only, and vape and tobacco) were self-reported in the follow-up questionnaire of 2019. If any cardiovascular disease or cancer were self-declared since their inclusion till 2019, participants were considered suffering from cancer and or cardiovascular disease.

### Statistical Analyses

The CONSTANCES cohort makes it possible to perform weighted prevalence analyses which provide results representative of the French general population covered by the general health insurance scheme [20]. Briefly, a weighting coefficient has been computed for each participant based on both the survey weight and the non-participation correction factor based on the follow-up of a control cohort of non-participants. The computation of the non-participation correction factor uses medico-administrative data from a control cohort of non-participants (more than 400,000 subjects). A detailed description of the methodology of computation of weighted analyses in the CONSTANCES cohort is available elsewhere and this methodology has been approved by the French authority that guarantees representativeness for the general population (Label du Conseil National de l’Information Statistique, CNIS) [20]. Since the data of the control cohort of non-participants come from medico-administrative registries, a delay is necessary before these data can be processed. In addition, in order to adjust for non-response to the 2019 follow-up questionnaire, an additional participation weight for this follow-up was computed. The product of both weights provided the final weight [21].

Since the level of alcohol consumption substantially differs between men and women, weighted prevalence rates of at-risk alcohol consumption were computed in both men and women and presented as percentages with a 95% confidence interval [2]. Weighted prevalence of exceeding each of the three indicators was also computed (i.e., exceeding two drinks per day, exceeding 10 drinks per week, and not having at least two alcohol-free days per week). Then, these analyses of weighted prevalence were then stratified for each sociodemographic and clinical factor. Finally, weighted odds ratios of at-risk alcohol consumption were computed with their 95% confidence interval in both men and women using two-sided binomial univariate logistic regressions for complex samples. In these logistic regressions, having an at-risk alcohol consumption was introduced as the binary dependent variable and each sociodemographic and clinical factor was introduced successively as the independent variable of interest. Multivariate analyses were not carried out, as the aim was to perform weighted descriptive analyses for public health purposes rather than epidemiological analyses to study the links between the variables measured.

Among the participants included in the present study, the prevalence of missing data on covariables ranged from 1.5% for education to 9.2% for having children, with a mean percentage of missing data of 3.1%. Assuming a missing at-random mechanism, multiple imputation was used to handle missing data [22]. Among the 34,470 participants, 1,107 participants (3.2%) had missing data on all alcohol consumption variables while 543 (1.6%) had missing data for at least one alcohol consumption variable. For these participants, we imputed the number of drinks per day.

In exploratory analyses, the estimates of exceeding the previous low-risk drinking guidelines (i.e., not exceeding two drinks per day for women, three drinks per day for men, four drinks per occasion, and having at least 1 day without alcohol consumption) were calculated to provide a comparison of the estimates based on the new and previous guidelines [12].

Finally, as sensitivity analyses, the main analyses were again conducted in a subgroup of participants without missing data, i.e., complete-case analysis (*n* = 32,820; 95.2% of the entire sample).

All the analyses were conducted with IBM SPSS Statistics for Windows, Version 21.0. Armonk, NY: IBM Corp.

## Results

In 2019, 47.8% (95%CI: 47.0%–48.5%) of the French general population exceeded at least one of the three criteria of low-risk alcohol consumption. Exceeding at least one of those criteria affected more men than women, i.e., 60.3% (95%CI: 59.2%–61.4%) and 36.4% (95%CI: 35.4%–37.4%) in men and women respectively (Tables 1, 2). In men, 49.2% (95%CI: 48.1%–50.3%) exceeded two drinks per day, 38.0% (95%CI: 36.9%–39.1%) exceeded 10 drinks per week, and 41.6% (95%CI: 40.5%–42.7%) had less than two alcohol-free days per week. In women, 36.4% (95%CI: 35.4%–37.4%) exceeded two drinks per day, 17.8% (95%CI: 17.0%–18.6%) exceeded 10 drinks per week, and 22.6% (95%CI: 21.8%–23.5%) had less than two alcohol-free days per week. These prevalence, stratified by sociodemographic and clinical factors, are presented in Supplementary Tables S1–S6.

**TABLE 1:** Sociodemographic characteristics of the population exceeding at least one of the three criteria from the low-risk drinking guidelines according to gender in 2019 (*N*, weighted percentages and 95% confidence intervals, *n* = 34,470) (The CONSTANCES study, Metropolitan France, 2019).

	Men		Women	
	*N* = 15,369		*N* = 19,101	
	Exceeding the recommendations		Exceeding the recommendations	
	9,251	60.3% (59.2%–61.4%)	6,872	36.4% (35.4%–37.4%)
Age				
20–34	1,434	58.6% (56.0%–61.1%)	1,236	38.4% (36.3%–40.6%)
35–44	1,975	58.8% (56.6%–61.0%)	1,607	35.1% (33.2%–37.0%)
45–54	1,970	57.3% (55.0%–59.5%)	1,424	33.9% (31.9%–35.9%)
55–64	2,059	61.4% (58.9%–63.8%)	1,414	35.7% (33.6%–37.9%)
65–74	1,813	66.6% (64.0%–69.2%)	1,191	39.2% (36.7%–41.8%)
Employment status				
Employed	6,087	58.7% (57.4%–60.0%)	4,595	35.4% (34.3%–36.5%)
Unemployed	545	56.4% (52.3%–60.4%)	514	36.4% (33.1%–39.9%)
Retired	2,545	66.5% (64.2%–68.6%)	1,661	38.7% (36.6%–40.9%)
Student	74	54.0% (43.2%–64.5%)	102	42.8% (35.1%–50.8%)
Education^a^				
Level 0 and Level 1	226	60.4% (54.0%–66.5%)	114	37.4% (30.8%–44.5%)
Level 2	450	63.3% (58.4%–67.9%)	352	40.0% (35.8%–44.4%)
Level 3 and Level 4	2,896	59.7% (57.8%–61.6%)	1,709	33.4% (31.7%–35.2%)
Level 5 and Level 6	2,946	60.3% (58.3%–62.2%)	2,829	35.4% (33.9%–36.8%)
Level 7 and Level 8	2,733	60.5% (58.5%–62.4%)	1,868	41.8% (39.9%–43.8%)
Occupational Grade				
Never worked	95	51.8% (42.6%–60.8%)	110	38.2% (31.2%–45.8%)
Manual worker or employee	3,005	59.3% (57.5%–61.1%)	2,504	34.1% (32.7%–35.6%)
Intermediate profession	2,298	59.5% (57.2%–61.6%)	2,232	35.3% (33.7%–37.1%)
Executive	3,853	62.7% (61.0%–64.4%)	2,023	43.0% (41.1%–44.9%)
Household Income				
<2100 euros	1,611	56.8% (54.4%–59.2%)	1,441	36.8% (34.8%–38.8%)
(2100–2800)	1,319	60.7% (57.9%–63.4%)	1,062	32.9% (30.7%–35.3%)
(2800–4200)	3,136	60.7% (58.8%–62.5%)	2,244	35.5% (33.9%–37.1%)
≥4200	3,185	63.4% (61.6%–65.3%)	2,125	40.2% (38.4%–42.0%)
Marital Status				
Single	3,067	59.2% (57.4%–61.1%)	2,772	38.2% (36.7%–39.8%)
In couple	6,184	61.0% (59.7%–62.4%)	4,100	35.0% (33.8%–36.3%)
Children				
Yes	5,591	60.8% (59.4%–62.2%)	4,644	34.6% (33.5%–35.8%)
No	3,660	59.7% (58.1%–61.4%)	2,228	39.6% (37.8%–41.3%)

^a^Based on the International Standard Classification of Education.

**TABLE 2:** Clinical characteristics of the population exceeding at least one of the three criteria from the low-risk drinking guidelines according to gender in 2019 (*N*, weighted percentages and 95% confidence intervals, *n* = 34,470) (The CONSTANCES study, Metropolitan France, 2019).

	Men		Women	
	*N* = 15,369		*N* = 19,101	
	Exceeding the recommendations		Exceeding the recommendations	
	9,251	60.3% (59.2%–61.4%)	6,872	36.4% (35.4%–37.4%)
Pregnant				
Yes			27	6.7% (4.1%–10.6%)
No			6,845	37.1% (36.2%–38.1%)
ERI^a^				
<1	6,093	61.5% (60.1%–62.8%)	4,299	37.0% (35.8%–38.3%)
1	183	69.3% (61.8%–76.0%)	117	39.2% (32.2%–46.7%)
>1	2,975	57.4% (55.5%–59.2%)	2,456	35.1% (33.6%–36.7%)
Treated Depression				
Yes	301	52.6% (47.0%–58.2%)	406	41.6% (37.4%–46.0%)
No	8,950	60.7% (59.6%–61.8%)	6,466	36.0% (35.0%–37.0%)
AUDIT Dependence score^b^				
0	4,872	52.5% (51.0%–53.9%)	4,362	30.9% (29.8%–32.0%)
[1–2]	2,319	66.8% (64.6%–69.0%)	1,562	47.2% (44.8%–49.6%)
≥3	2,060	75.3% (72.9%–77.6%)	948	56.5% (53.2%–59.7%)
Binge Drinking				
Never	3,999	48.6% (47.1%–50.2%)	4,367	30.1% (29.1%–31.2%)
At least once	4,973	73.4% (72.0%–74.8%)	2,219	57.5% (55.4%–59.6%)
Cannabis use				
Not during the previous 12 months	8,274	58.4% (57.2%–59.5%)	6,392	34.8% (33.8%–35.8%)
At least once during the previous 12 months	977	77.9% (74.7%–80.8%)	480	67.8% (62.8%–72.4%)
Smoking Status				
Non-smoker or occasional smoker	8,004	58.5% (57.3%–59.6%)	5,818	33.9% (32.9%–34.9%)
Smoker	1,247	71.0% (68.0%–73.8%)	1,054	52.8% (49.7%–55.8%)
Smoking and Vaping				
No use	7,701	58.1% (56.9%–59.2%)	5,636	33.7% (32.7%–34.7%)
Vape only	303	68.2% (61.9%–74.0%)	182	40.8% (34.7%–47.2%)
Tobacco only	1,060	70.2% (66.9%–73.3%)	930	53.3% (50.0%–56.5%)
Vape and tobacco	187	75.0% (67.4%–81.2%)	124	49.9% (41.5%–58.3%)
Self-rated Health^c^				
[1–3]	7,780	61.1% (59.9%–62.3%)	5,725	36.5% (35.5%–37.6%)
>3	1,471	57.4% (54.7%–60.0%)	1,147	35.8% (33.5%–38.1%)
CVD^d^				
Yes	1,743	60.3% (57.7%–62.9%)	872	34.7% (32.0%–37.5%)
No	7,508	60.3% (59.1%–61.5%)	6,000	36.7% (35.7%–37.7%)
Cancer				
Yes	275	54.6% (48.1%–60.9%)	183	31.5% (26.3%–37.2%)
No	8,976	60.6% (59.5%–61.7%)	6,689	36.6% (35.6%–37.5%)

^a^ Effort-reward imbalance. <1, indicating an imbalance in favor of reward; 1, effort-reward balance,; >1, indicating an imbalance in favor of effort.

^b^ Alcohol Use Disorder Identification Test, items 3 to 10.

^c^ Self-rated health from 1 = “very good” to 8 = “very poor.”

^d^ Cardiovascular disease.

### Sociodemographic Factors

Exceeding the guidelines was more common among older (65–74 years old) compared to younger men OR 1.41 95%CI (1.21–1.65). In women, the patterns were different with low rates among those aged (35–44 years) OR 0.86 95%CI (0.76–0.98) and (45–54 years) OR 0.82 95%CI (0.72–0.93) compared to younger women (20–34 years old).

Retired men and women were more likely to exceed the low-risk drinking guidelines compared to employed individuals with OR 1.39 95%CI (1.24–1.56) and OR 1.15 95%CI (1.04–1.28), respectively.

Men with an occupational grade of executive were more likely to exceed the low-risk drinking guidelines compared to men who have never worked with OR 1.56 95%CI (1.07–2.28).

Compared to a household income <2,100 euros, men with a household income of 2,100–2,800 euros, 2,800–4,200 euros, or ≥4,200 euros were more likely to exceed the guidelines with OR 1.17 95%CI (1.01–1.36), OR 1.17 95%CI (1.03–1.32), and OR 1.32 95%CI (1.16–1.50), respectively and with a significant linear trend (*p* < 0.001). Among women, only those with a household income ≥4,200 euros were significantly more likely to exceed the guidelines than women with a household income <2,100 euros OR 1.15 95%CI (1.02–1.29) while women with a household income of 2,100–2,800 euros were less likely to exceed the guidelines with OR 0.84 95%CI (0.73–0.96).

Women who has a partner were less likely to exceed the guidelines than single women with an OR 0.87 95%CI (0.80–0.95), and the guidelines were exceeded more by women with no children than women who have children with an OR 1.23 95%CI (1.13–1.34) (Table 3).

**TABLE 3:** Odds of exceeding at least one of the three criteria from the low-risk drinking guidelines according to each sociodemographic determinant in 2019 (weighted univariate logistic regression, OR and 95% confidence interval, *n* = 34,470) (The CONSTANCES study, Metropolitan France, 2019).

	Exceeding at least one of the recommendations	
	Men	Women
	OR 95%CI	OR 95%CI
Age		
20–34	Ref	Ref
35–44	1.01 (0.88–1.16)	**0.86 (0.76–0.98)**
45–54	0.94 (0.82–1.08)	**0.82 (0.72–0.93)**
55–64	1.12 (0.97–1.30)	0.89 (0.78–1.01)
65–76	**1.41 (1.21–1.65)**	1.03 (0.90–1.19)
Employment status		
Employed	Ref	Ref
Unemployed	0.91 (0.76–1.08)	1.04 (0.89–1.22)
Retired	**1.39 (1.24–1.56)**	**1.15 (1.04–1.28)**
Student	0.82 (0.53–1.28)	1.36 (0.98–1.89)
Education ^a^		
Level 0 and Level 1	Ref	Ref
Level 2	1.12 (0.81–1.57)	1.11 (0.79–1.57)
Level 3 and Level 4	0.97 (0.74–1.27)	0.84 (0.61–1.14)
Level 5 and Level 6	0.99 (0.75–1.30)	0.91 (0.67–1.24)
Level 7 and Level 8	1.00 (0.76–1.32)	1.20 (0.88–1.63)
Occupational Grade		
Never worked	Ref	Ref
Manual worker or employee	1.35 (0.93–1.98)	0.84 (0.60–1.15)
Intermediate profession	1.36 (0.93–2.00)	0.88 (0.64–1.21)
Executive	**1.56 (1.07–2.28)**	1.22 (0.88–1.68)
Household Income		
<2,100 euros	Ref	Ref
(2,100–2,800)	**1.17 (1.01–1.36)**	**0.84 (0.73–0.96)**
(2,800–4,200)	**1.17 (1.03–1.32)**	0.94 (0.84–1.05)
≥4,200	**1.32 (1.16–1.50)**	**1.15 (1.02–1.29)**
Marital Status		
Single	Ref	Ref
In couple	1.07 (0.98–1.18)	**0.87 (0.80–0.95)**
Children		
Yes	Ref	Ref
No	0.95 (0.87–1.04)	**1.23 (1.13–1.34)**

^a^ Based on the International Standard Classification of Education.

Bold values represents a *p* value <0.005.

### Clinical Factors

Exceeding the low-risk drinking guidelines was less common among pregnant women OR 0.12 95%CI (0.07–0.20).

Exceeding the guidelines was more common among men with no ERI with OR 1.13 95%CI (1.04–1.24) and less common for men with an ERI in favor of effort with OR 0.84 95%CI (0.76–0.92) than those with an ERI in favor of reward.

Exceeding the low-risk drinking guidelines was less common among men who were treated for depression OR 0.71 95%CI (0.57–0.90) while it was more common among women treated for depression OR 1.26 95%CI (1.05–1.52) compared to those who were not.

Compared to an AUDIT dependence score of 0, men and women with a score of 1–2 were more likely to exceed the guidelines OR 1.82 95%CI (1.63–2.05) and 1.99 95%CI (1.79–2.22) respectively; as well, men and women with a score ≥3 OR 2.76 95%CI (2.41–3.17) and OR 2.89 95%CI (2.51–3.34), respectively. A dose-dependent relationship was observed among men and women (*p* < 0.001). Men and women who experienced binge drinking at least once during the previous 12 months were more likely to exceed the guidelines than those who did not with OR 2.91 95%CI (2.65–3.21) and OR 3.13 95%CI (2.84–3.46), respectively.

Men and women who consumed cannabis during the previous 12 months were more likely to exceed the guidelines than those who did not with OR 2.51 95%CI (2.09–3.02) and OR 3.94 95%CI (3.15–4.94), respectively.

Among both men and women, smokers were more likely to exceed the low-risk drinking guidelines than non-smokers or occasional smokers with OR 1.74 95%CI (1.50–2.02) and OR 2.17 95%CI (1.91–2.47), respectively. Additionally, compared to no use of tobacco nor vaping, exceeding the guidelines was significantly more common among men and women who only vape, who only smoke, or those who vape and smoke with a significant trend (*p* < 0.001) for men.

Men with poorer self-rated health were less likely to exceed the guidelines OR 0.85 95%CI (0.76–0.96). However, the likelihood of exceeding the guidelines was not found to be related to having a cardiovascular disease or cancer (Table 4).

**TABLE 4:** Odds of exceeding at least one of the three criteria from the low-risk drinking guidelines according to each clinical determinant in 2019 (weighted univariate logistic regression, OR and 95% confidence interval, *n* = 34,470) (The CONSTANCES study, Metropolitan France, 2019).

	Exceeding at least one of the recommendations	
	Men	Women
	OR 95%CI	OR 95%CI
Pregnant		
No		Ref
Yes		**0.12 (0.07–0.20)**
ERI^a^		
<1	Ref	Ref
1	**1.41 (1.01–1.99)**	1.09 (0.80–1.49)
>1	**0.84 (0.76–0.92)**	0.92 (0.84–1.01)
Treated Depression		
No	Ref	Ref
Yes	**0.71 (0.57–0.90)**	**1.26 (1.05–1.52)**
AUDIT Dependence score^b^		
0	Ref	Ref
(1–2)	**1.82 (1.63–2.05)**	**1.99 (1.79–2.22)**
≥3	**2.76 (2.41–3.17)**	**2.89 (2.51–3.34)**
Binge Drinking		
Never	Ref	Ref
At least once	**2.91 (2.65–3.21)**	**3.13 (2.84–3.46)**
Cannabis use		
Not during the previous 12 months	Ref	Ref
At least once during the previous 12 months	**2.51 (2.09–3.02)**	**3.94 (3.15–4.94)**
Smoking Status		
Non-smoker or occasional smoker	Ref	Ref
Smoker	**1.74 (1.50–2.02)**	**2.17 (1.91–2.47)**
Smoking and Vaping		
No use	Ref	Ref
Vape only	**1.55 (1.16–2.06)**	**1.35 (1.03–1.76)**
Tobacco only	**1.70 (1.45–1.99)**	**2.24 (1.95–2.57)**
Vape and tobacco	**2.16 (1.48–3.13)**	**1.95 (1.39–2.76)**
Self-rated Health^c^		
[1–3]	Ref	Ref
>3	**0.85 (0.76–0.96)**	0.96 (0.86–1.08)
CVD^d^		
No	Ref	Ref
Yes	0.99 (0.88–1.12)	0.91 (0.80–1.04)
Cancer		
No	Ref	Ref
Yes	0.78 (0.60–1.02)	0.79 (0.61–1.03)

^a^ Effort-reward imbalance. <1, indicating an imbalance in favor of reward; 1, effort-reward balance; >1, indicating an imbalance in favor of effort.

^b^ Alcohol Use Disorder Identification Test, items 3 to 10.

^c^ Self-rated health from 1 = “very good” to 8 = “very poor.”

^d^ Cardiovascular disease.

Bold values represents a *p* value <0.005.

Under the new guidelines, 47.8% (95%CI: 47.0%–48.5%) of the French general population exceeded levels of at-risk alcohol, compared to 40.1% (95%CI: 39.4%–40.9%) under the previous 2019 guidelines. Among men, 60.3% (95%CI: 59.2%–61.4%) exceeded the new guidelines, while 47.7% (95%CI: 46.6%–48.8%) exceeded the previous guidelines (i.e., an increase of 20.8%). Among women, 36.4% (95%CI: 35.4%–37.4%) exceeded the new guidelines, while 33.3% (95%CI: 32.3%–34.2%) exceeded the previous guidelines (i.e., an increase of 8.5%).

After excluding participants with missing data for alcohol use, similar weighted prevalence was found in both men and women (Supplementary Tables S7, S8).

## Discussion

Our aim was to estimate the proportion of the participants of the French national population-based CONSTANCES cohort who exceed the new national guidelines of low-risk drinking (i.e., no more than 10 standard drinks per week, no more than two standard drinks per day, and at least two alcohol-free days every week) [11]. In 2019, almost 48% of these participants from the French general population exceeded these guidelines (60% in men and 37% in women). In both genders, the criterion of two standard drinks per day was the one that was the most frequently exceeded. The criteria of socioeconomic status, smoking, vaping, using cannabis, and having experienced heavy episodic drinking or alcohol dependence in the preceding 12 months were related to an increased likelihood of exceeding the guidelines. In men, being older or having no ERI compared to an ERI in favor of reward was related to an increased likelihood of exceeding the guidelines, while having a poorer self-rated health status, being depressed, or having an ERI in favor of effort were related to decreased likelihoods of exceeding the guidelines. In women, being younger or depressed was related to an increased likelihood of exceeding the guidelines. Almost 8% of the pregnant women exceeded the low-drinking guidelines.

To compute the estimates of prevalence, we used data from a national population-based cohort in which participants were randomly recruited at enrollment and came from various regions of France [11]. In addition, estimates were weighted to account for selection biases and ensure representativeness for the French general population [20]. Finally, a broad range of sociodemographic and clinical factors could be examined. However, this study has several limitations. First, even if participants were randomly selected and the prevalence was weighted, we cannot exclude that the estimates could be still affected by the propensity of participants in an epidemiologic cohort to be healthier and low-risk drinkers [23]. Second, participants from the CONSTANCES cohort are limited to those who were 18–69 years at enrollment (with the oldest participant included in the present study being 74 years of age), affiliated with the general insurance scheme (covering 93% of the French population), and living in metropolitan regions (i.e., excluding overseas). Thus, representativeness of the estimates is restricted to this population, and extrapolations to other groups should be done with great caution. In particular, studies among younger and older subjects should be conducted, as well as among people with other insurance schemes or without insurance (e.g., foreigners living in France, undocumented migrants). Third, in the CONSTANCES cohort, we did not have data for the number of drinks separately for each day from Monday to Thursday which may have resulted in overestimating participants exceeding the third criterion (at least two alcohol-free days per week). Fourth, since the data on alcohol consumption was self-reported, they could be subject to social desirability bias. However, confidential surveys were found to be one of the methods least associated with social desirability bias for self-reported health risk behaviors [24, 25]. Fifth, a standard drink in France corresponds to approximately 10 g of pure alcohol and the thresholds used to establish the French guidelines of low-risk drinking should be considered in the light of this definition.

The prevalence of exceeding the low-risk drinking guidelines was in line with previous findings from other countries such as Australia and the United Kingdom, although with mostly higher benchmarks for alcohol use [26–28]. The higher prevalence of exceeding the guidelines in men, older participants, and those from higher socioeconomic backgrounds were as well in line with other findings from France as well as from other countries [1, 2, 26, 28]. These associations may be explained by a generational effect (i.e., overall decrease in alcohol consumption in younger generations) and more opportunities to consume alcohol for people from higher socioeconomic backgrounds due to their wider social circle, lack of financial barriers, and better health status [2, 29, 30].

Surprisingly, among men, work stress was negatively associated with exceeding the low-risk drinking guidelines in the present study contradicting other studies that found an increased risk of alcohol use according to work stress. However, these prior studies considered high levels of consumption and/or alcohol-related harms rather than national low-risk drinking guidelines [17, 31]. In addition, a better socioeconomic status is associated with a better quality of employment while work stress is more prevalent among individuals with a lower socioeconomic status [32]. Thus, the association between work stress and alcohol use needs to be further adjusted for socioeconomic status. Treated depression was associated with a higher likelihood of exceeding the guideline in women, while the opposite was true in men. The interpretation of this result is not obvious and would require further exploration. However, this finding is in line with some studies showing gender differences regarding the associations between alcohol consumption and depression or depressive state [33, 34]. These results might be due to different strategies between men and women to cope with depressive symptoms [35]. Moreover, although for pregnant women, the recommendations are to abstain from drinking, almost 8% of the pregnant women exceeded the guidelines for the general population. Thus, information and prevention campaigns should be more focused on the medical risk to the unborn child, and Screening, Brief Intervention, and Referral to Treatment (SBIRT) should be reinforced as soon as there is a desire to become pregnant, and then throughout the pregnancy [2, 36]. Although a lower prevalence of exceeding the guidelines among men with poor self-rated health was observed, this was not the case for cardiovascular diseases and cancers although alcohol is associated with a poorer prognosis in patients suffering from these disorders [2]. Therefore, reinforced public health awareness campaigns regarding the adverse consequences of alcohol on health and the implementation of effective prevention measures among individuals with cardiovascular diseases or cancers may be needed.

Alcohol consumption frequently co-occurs with the use of other substances such as tobacco and cannabis [1]. The co-use of these substances increases the perceived rewarding effects of each substance and a « cross-tolerance » effect may lead to higher needs for these substances [37, 38]. Notably, electronic cigarette users had a lower prevalence of at-risk drinking compared to smokers. Since most adult electronic cigarette users are former smokers [39], one could hypothesize that electronic cigarette use could have some benefits among former smokers on the level of alcohol consumption. Having a history of binge drinking or having criteria of alcohol dependence were found to be strong indicators of having at-risk alcohol consumption [40]. Reinforced prevention strategies and early detection of these predictors of at-risk alcohol use are of great importance.

To conclude, our findings highlight the importance of intensifying the public health awareness of the French population regarding the harm of alcohol use and the new low-risk drinking guidelines, especially among men, individuals with high socioeconomic status, individuals with health problems, retired individuals, smokers, and cannabis users. It would also be important to step up screening for at-risk alcohol consumption that can be carried out at every contact with primary care. Thus, all caregivers must be trained in SBIRT [2]. Furthermore, pursuing public policies to prevent at-risk consumption by controlling the supply should be reinforced [2]. Future studies should assess the prevalence of exceeding low-risk drinking guidelines during the COVID pandemic and compare them with the pre-pandemic period. Finally, the design of the present study, which was focused on the calculation of prevalence, was not suited to epidemiological analyses (e.g., multivariate analyses including moderation and/or mediation models). Thus, future studies with a longitudinal follow-up should explore the underlying mechanisms leading to the associations between at-risk alcohol consumption and sociodemographic and clinical factors.
