# Supplementary material for: Proportion of At-Risk Alcohol Consumers According to the New French Guidelines: Cross-Sectional Weighted Analyses From the CONSTANCES Cohort
Source: Int J Public Health. 2024 Feb 16;69:1606481. doi: 10.3389/ijph.2024.1606481 (PMC10904535; doi:10.3389/ijph.2024.1606481)
Supplement: Supplementary file 1 [file DataSheet1.docx]

Supplemental Table 1. Sociodemographic characteristics of the population exceeding 2 drinks per day according to gender in 2019. (weighted percentages and 95% confidence intervals, n=34,470) (The CONSTANCES study, Metropolitan France, 2019)

|  | **Not exceeding 2 drinks per day** | | | |
| --- | --- | --- | --- | --- |
|  | **Men** | | **Women** | |
|  | **Not exceeding the recommendations** | **Exceeding the recommendations** | **Not exceeding the recommendations** | **Exceeding the recommendations** |
|  | 8 009 50.8% (49.7%-51.9%) | 7 360 49.2% (48.1%-50.3%) | 14 179 72.8% (71.9%-73.7%) | 4 922 27.2% (26.3%-28.1%) |
| **Age** |  |  |  |  |
| 20-34 | 49.4% (46.9%-52.0%) | 50.6% (48.0%-53.1%) | 67.6% (65.5%-69.6%) | 32.4% (30.4%-34.5%) |
| 35-44 | 50.7% (48.4%-52.9%) | 49.3% (47.1%-51.6%) | 73.5% (71.6%-75.2%) | 26.5% (24.8%-28.4%) |
| 45-54 | 52.9% (50.6%-55.2%) | 47.1% (44.8%-49.4%) | 74.1% (72.2%-76.0%) | 25.9% (24.0%-27.8%) |
| 55-64 | 49.9% (47.4%-52.4%) | 50.1% (47.6%-52.6%) | 75.0% (72.9%-76.9%) | 25.0% (23.1%-27.1%) |
| 65-74 | 50.9% (48.2%-53.7%) | 49.1% (46.3%-51.8%) | 75.7% (73.3%-77.9%) | 24.3% (22.1%-26.7%) |
| **Employment status** |  |  |  |  |
| Employed | 51.2% (49.9%-52.5%) | 48.8% (47.5%-50.1%) | 72.5% (71.5%-73.6%) | 27.5% (26.4%-28.5%) |
| Unemployed | 50.4% (46.3%-54.5%) | 49.6% (45.5%-53.7%) | 72.1% (68.8%-75.1%) | 27.9% (24.9%-31.2%) |
| Retired | 49.4% (47.1%-51.7%) | 50.6% (48.3%-52.9%) | 75.1% (73.1%-77.0%) | 24.9% (23.0%-26.9%) |
| Student | 55.3% (44.5%-65.6%) | 44.7% (34.4%-55.5%) | 61.6% (53.5%-69.1%) | 38.4% (30.9%-46.5%) |
| **Education ^a^** |  |  |  |  |
| Level 0 and Level 1 | 46.5% (40.2%-53.0%) | 53.5% (47.0%-59.8%) | 69.5% (62.5%-75.7%) | 30.5% (24.3%-37.5%) |
| Level 2 | 46.4% (41.5%-51.3%) | 53.6% (48.7%-58.5%) | 71.2% (67.0%-75.0%) | 28.8% (25.0%-33.0%) |
| Level 3 and Level 4 | 51.3% (49.4%-53.2%) | 48.7% (46.8%-50.6%) | 74.5% (72.8%-76.2%) | 25.5% (23.8%-27.2%) |
| Level 5 and Level 6 | 51.2% (49.2%-53.2%) | 48.8% (46.8%-50.8%) | 74.1% (72.7%-75.4%) | 25.9% (24.6%-27.3%) |
| Level 7 and Level 8 | 51.3% (49.3%-53.3%) | 48.7% (46.7%-50.7%) | 68.5% (66.6%-70.3%) | 31.5% (29.7%-33.4%) |
| **Occupational Grade** |  |  |  |  |
| Never worked | 52.8% (43.6%-61.8%) | 47.2% (38.2%-56.4%) | 65.6% (58.1%-72.5%) | 34.4% (27.5%-41.9%) |
| Manual worker or employee | 50.2% (48.4%-52.0%) | 49.8% (48.0%-51.6%) | 73.9% (72.6%-75.3%) | 26.1% (24.7%-27.4%) |
| Intermediate profession | 53.9% (51.6%-56.1%) | 46.1% (43.9%-48.4%) | 75.5% (74.0%-77.0%) | 24.5% (23.0%-26.0%) |
| Executive | 49.4% (47.7%-51.1%) | 50.6% (48.9%-52.3%) | 67.4% (65.6%-69.2%) | 32.6% (30.8%-34.4%) |
| **Household Income** |  |  |  |  |
| <2100 euros | 51.6% (49.2%-54.0%) | 48.4% (46.0%-50.8%) | 70.5% (68.5%-72.4%) | 29.5% (27.6%-31.5%) |
| [2100-2800[ | 51.2% (48.4%-54.0%) | 48.8% (46.0%-51.6%) | 76.1% (74.0%-78.1%) | 23.9% (21.9%-26.0%) |
| [2800-4200[ | 51.3% (49.4%-53.1%) | 48.7% (46.9%-50.6%) | 74.2% (72.6%-75.7%) | 25.8% (24.3%-27.4%) |
| ≥4200 | 48.9% (47.0%-50.8%) | 51.1% (49.2%-53.0%) | 71.1% (69.4%-72.8%) | 28.9% (27.2%-30.6%) |
| **Marital Status** |  |  |  |  |
| Single | 50.0% (48.1%-51.8%) | 50.0% (48.2%-51.9%) | 70.1% (68.6%-71.6%) | 29.9% (28.4%-31.4%) |
| In couple | 51.3% (49.9%-52.7%) | 48.7% (47.3%-50.1%) | 74.8% (73.7%-75.9%) | 25.2% (24.1%-26.3%) |
| **Children** |  |  |  |  |
| Yes | 51.5% (50.1%-53.0%) | 48.5% (47.0%-49.9%) | 75.1% (74.0%-76.1%) | 24.9% (23.9%-26.0%) |
| No | 49.9% (48.1%-51.6%) | 50.1% (48.4%-51.9%) | 68.7% (67.0%-70.3%) | 31.3% (29.7%-33.0%) |
| ^a^ Based on the International Standard Classification of Education. | | | | |
|  |  |  |  |  |

Supplemental Table 2. Clinical characteristics of the population exceeding 2 drinks per day according to gender in 2019. (weighted percentages and 95% confidence intervals, n=34,470) (The CONSTANCES study, Metropolitan France, 2019)

|  | **Not exceeding 2 drinks per day** | | | |
| --- | --- | --- | --- | --- |
|  | **Men** | | **Women** | |
|  | **Not exceeding the recommendations** | **Exceeding the recommendations** | **Not exceeding the recommendations** | **Exceeding the recommendations** |
|  | 8 009 50.8% (49.7%-51.9%) | 7 360 49.2% (48.1%-50.3%) | 14 179 72.8% (71.9%-73.7%) | 4 922 27.2% (26.3%-28.1%) |
| **Pregnant** |  |  |  |  |
| Yes |  |  | 95.6% (92.0%-97.6%) | 4.4% (2.4%-8.0%) |
| No |  |  | 72.2% (71.3%-73.1%) | 27.8% (26.9%-28.7%) |
| **ERI ^a^** |  |  |  |  |
| <1 | 49.8% (48.5%-51.2%) | 50.2% (48.8%-51.5%) | 72.8% (71.6%-74.0%) | 27.2% (26.0%-28.4%) |
| 1 | 49.1% (41.1%-57.1%) | 50.9% (42.9%-58.9%) | 71.7% (64.3%-78.0%) | 28.3% (22.0%-35.7%) |
| >1 | 52.9% (51.0%-54.7%) | 47.1% (45.3%-49.0%) | 72.8% (71.3%-74.3%) | 27.2% (25.7%-28.7%) |
| **Treated Depression** |  |  |  |  |
| Yes | 56.5% (50.8%-62.0%) | 43.5% (38.0%-49.2%) | 66.3% (62.0%-70.3%) | 33.7% (29.7%-38.0%) |
| No | 50.5% (49.3%-51.6%) | 49.5% (48.4%-50.7%) | 73.3% (72.4%-74.2%) | 26.7% (25.8%-27.6%) |
| **AUDIT Dependence score ^b^** |  |  |  |  |
| 0 | 60.1% (58.7%-61.5%) | 39.9% (38.5%-41.3%) | 78.5% (77.5%-79.5%) | 21.5% (20.5%-22.5%) |
| [1-2] | 43.5% (41.2%-45.8%) | 56.5% (54.2%-58.8%) | 61.9% (59.6%-64.2%) | 38.1% (35.8%-40.4%) |
| ≥3 | 32.3% (29.9%-34.8%) | 67.7% (65.2%-70.1%) | 51.1% (47.8%-54.4%) | 48.9% (45.6%-52.2%) |
| **Binge Drinking** |  |  |  |  |
| Never | 63.7% (62.3%-65.2%) | 36.3% (34.8%-37.7%) | 79.1% (78.2%-80.0%) | 20.9% (20.0%-21.8%) |
| At least once | 36.2% (34.6%-37.7%) | 63.8% (62.3%-65.4%) | 51.5% (49.4%-53.6%) | 48.5% (46.4%-50.6%) |
| **Cannabis use** |  |  |  |  |
| Not during the previous 12 months | 52.9% (51.8%-54.1%) | 47.1% (45.9%-48.2%) | 74.5% (73.6%-75.4%) | 25.5% (24.6%-26.4%) |
| At least once during the previous 12 months | 31.3% (28.0%-34.8%) | 68.7% (65.2%-72.0%) | 39.9% (35.0%-44.9%) | 60.1% (55.1%-65.0%) |
| **Smoking Status** |  |  |  |  |
| Non-smoker or occasional smoker | 52.9% (51.7%-54.1%) | 47.1% (45.9%-48.3%) | 75.0% (74.1%-75.9%) | 25.0% (24.1%-25.9%) |
| Smoker | 38.5% (35.5%-41.6%) | 61.5% (58.4%-64.5%) | 58.0% (55.0%-61.0%) | 42.0% (39.0%-45.0%) |
| **Smoking and Vaping** |  |  |  |  |
| No use | 53.2% (52.0%-54.4%) | 46.8% (45.6%-48.0%) | 75.3% (74.4%-76.2%) | 24.7% (23.8%-25.6%) |
| Vape only | 44.4% (38.1%-50.9%) | 55.6% (49.1%-61.9%) | 67.5% (59.2%-71.2%) | 34.5% (28.8%-40.8%) |
| Tobacco only | 39.3% (36.0%-42.7%) | 60.7% (57.3%-64.0%) | 57.8% (54.5%-61.0%) | 42.2% (39.0%-45.5%) |
| Vape and tobacco | 34.4% (27.3%-42.2%) | 65.6% (57.8%-72.7%) | 59.3% (50.8%-67.3%) | 40.7% (32.7%-49.2%) |
| **Self-rated Health ^c^** |  |  |  |  |
| [1-3] | 50.2% (49.0%-51.4%) | 49.8% (48.6%-51.0%) | 73.0% (72.0%-74.0%) | 27.0% (26.0%-28.0%) |
| >3 | 52.7% (50.0%-55.4%) | 47.3% (44.6%-50.0%) | 71.9% (69.7%-74.1%) | 28.1% (25.9%-30.3%) |
| **CVD ^d^** |  |  |  |  |
| Yes | 51.1% (48.4%-53.7%) | 48.9% (46.3%-51.6%) | 75.1% (72.4%-77.5%) | 24.9% (22.5%-27.6%) |
| No | 50.7% (49.5%-51.9%) | 49.3% (48.1%-50.5%) | 72.4% (71.4%-73.4%) | 27.6% (26.6%-28.6%) |
| **Cancer** |  |  |  |  |
| Yes | 57.8% (51.4%-64.0%) | 42.2% (36.0%-48.6%) | 77.8% (72.7%-82.3%) | 22.2% (17.7%-27.3%) |
| No | 50.4% (49.3%-51.6%) | 49.6% (48.4%-50.7%) | 72.6% (71.7%-73.5%) | 27.4% (26.5%-28.3%) |
| ^a^ Effort reward imbalance. (<1: indicating an imbalance in favor of reward, 1: effort reward balance, >1: indicating an imbalance in favor of effort.  ^b^ Alcohol Use Disorder Identification Test, item 3 to 10.  ^c^ Self-rated health from 1= “very good” to 8= “very poor”.  ^d^ Cardiovascular disease. | | | | |

Supplemental Table 3. Sociodemographic characteristics of the population exceeding 10 drinks per week according to gender in 2019. (weighted percentages and 95% confidence intervals, n=34,470) (The CONSTANCES study, Metropolitan France, 2019)

|  | **Not exceeding 10 drinks per week** | | | |
| --- | --- | --- | --- | --- |
|  | **Men** | | **Women** | |
|  | **Not exceeding the recommendations** | **Exceeding the recommendations** | **Not exceeding the recommendations** | **Exceeding the recommendations** |
|  | 9 701 62.0% (60.9%-63.1%) | 5 668 38.0% (36.9%-39.1%) | 15 947 82.2% (81.4%-83.0%) | 3 154 17.8% (17.0%-18.6%) |
| **Age** |  |  |  |  |
| 20-34 | 65.0% (62.5%-67.4%) | 35.0% (32.6%-37.5%) | 80.2% (78.4%-82.0%) | 19.8% (18.0%-21.6%) |
| 35-44 | 66.5% (64.4%-68.6%) | 33.5% (31.4%-35.6%) | 85.4% (83.9%-86.8%) | 14.6% (13.2%-16.1%) |
| 45-54 | 64.8% (62.5%-67.0%) | 35.2% (33.0%-37.5%) | 83.5% (81.7%-85.1%) | 16.5% (14.9%-18.3%) |
| 55-64 | 58.2% (55.7%-60.7%) | 41.8% (39.3%-44.3%) | 82.0% (80.1%-83.8%) | 18.0% (16.2%-19.9%) |
| 65-74 | 54.0% (51.3%-56.7%) | 46.0% (43.3%-48.7%) | 79.4% (77.2%-81.4%) | 20.6% (18.6%-22.8%) |
| **Employment status** |  |  |  |  |
| Employed | 65.1% (63.9%-66.3%) | 34.9% (33.7%-36.1%) | 83.4% (82.5%-84.3%) | 16.6% (15.7%-17.5%) |
| Unemployed | 59.3% (55.1%-63.3%) | 40.7% (36.7%-44.9%) | 80.7% (77.8%-83.4%) | 19.3% (16.6%-22.2%) |
| Retired | 54.6% (52.3%-57.0%) | 45.4% (43.0%-47.7%) | 79.7% (77.8%-81.4%) | 20.3% (18.6%-22.2%) |
| Student | 70.8% (60.4%-79.4%) | 29.2% (20.6%-39.6%) | 79.2% (71.5%-85.2%) | 20.8% (14.8%-28.5%) |
| **Education ^a^** |  |  |  |  |
| Level 0 and Level 1 | 53.5% (46.9%-59.9%) | 46.5% (40.1%-53.1%) | 75.8% (69.1%-81.4%) | 24.2% (18.6%-30.9%) |
| Level 2 | 56.9% (51.9%-61.7%) | 43.1% (38.3%-48.1%) | 80.3% (76.6%-83.5%) | 19.7% (16.5%-23.4%) |
| Level 3 and Level 4 | 62.1% (60.2%-63.9%) | 37.9% (36.1%-39.8%) | 83.5% (82.0%-84.9%) | 16.5% (15.1%-18.0%) |
| Level 5 and Level 6 | 62.3% (60.3%-64.1%) | 37.7% (35.9%-39.7%) | 83.5% (82.1%-84.9%) | 16.5% (15.1%-17.9%) |
| Level 7 and Level 8 | 64.4% (62.5%-66.4%) | 35.6% (33.6%-37.5%) | 79.2% (77.5%-80.7%) | 20.8% (19.3%-22.5%) |
| **Occupational Grade** |  |  |  |  |
| Never worked | 65.0% (55.8%-73.3%) | 35.0% (26.7%-44.2%) | 78.6% (71.6%-84.3%) | 21.4% (15.7%-28.4%) |
| Manual worker or employee | 61.4% (59.6%-63.2%) | 38.6% (36.8%-40.4%) | 83.0% (81.8%-84.1%) | 17.0% (15.9%-18.2%) |
| Intermediate profession | 63.5% (61.3%-65.6%) | 36.5% (34.4%-38.7%) | 83.3% (82.2%-84.2%) | 16.7% (15.8%-17.8%) |
| Executive | 61.8% (60.0%-63.4%) | 38.2% (36.6%-40.0%) | 80.7% (79.5%-81.7%) | 19.3% (18.3%-20.5%) |
| **Household Income** |  |  |  |  |
| <2100 euros | 60.5% (58.1%-62.8%) | 39.5% (37.2%-41.9%) | 80.2% (78.4%-81.8%) | 19.8% (18.2%-21.6%) |
| [2100-2800[ | 62.2% (59.4%-64.9%) | 37.8% (35.1%-40.6%) | 83.4% (81.4%-85.1%) | 16.6% (14.9%-18.6%) |
| [2800-4200[ | 63.6% (61.8%-65.4%) | 36.4% (34.6%-38.2%) | 83.8% (82.4%-85.0%) | 16.2% (15.0%-17.6%) |
| ≥4200 | 61.6% (59.7%-63.5%) | 38.4% (36.5%-40.3%) | 81.8% (80.2%-83.2%) | 18.2% (16.8%-19.8%) |
| **Marital Status** |  |  |  |  |
| Single | 60.5% (58.7%-62.3%) | 39.5% (37.7%-41.3%) | 80.3% (79.0%-81.6%) | 19.7% (18.4%-21.0%) |
| In couple | 63.0% (61.7%-64.3%) | 37.0% (35.7%-38.3%) | 83.6% (82.6%-84.6%) | 16.4% (15.4%-17.4%) |
| **Children** |  |  |  |  |
| Yes | 62.7% (61.3%-64.0%) | 37.3% (36.0%-38.7%) | 83.8% (82.9%-84.7%) | 16.2% (15.3%-17.1%) |
| No | 61.3% (59.6%-62.9%) | 38.7% (37.1%-40.4%) | 79.3% (77.8%-80.7%) | 20.7% (19.3%-22.2%) |
| ^a^ Based on the International Standard Classification of Education. | | | | |

Supplemental Table 4. Clinical characteristics of the population exceeding 10 drinks per week according to gender in 2019. (weighted percentages and 95% confidence intervals, n=34,470) (The CONSTANCES study, Metropolitan France, 2019)

|  | **Not exceeding 10 drinks per week** | | | |
| --- | --- | --- | --- | --- |
|  | **Men** | | **Women** | |
|  | **Not exceeding the recommendations** | **Exceeding the recommendations** | **Not exceeding the recommendations** | **Exceeding the recommendations** |
|  | 9 701 62.0% (60.9%-63.1%) | 5 668 38.0% (36.9%-39.1%) | 15 947 82.2% (81.4%-83.0%) | 3 154 17.8% (17.0%-18.6%) |
| **Pregnant** |  |  |  |  |
| Yes |  |  | 96.6% (93.0%-98.4%) | 3.4% (1.6%-7.0%) |
| No |  |  | 81.9% (81.0%-82.7%) | 18.1% (17.3%-19.0%) |
| **ERI ^a^** |  |  |  |  |
| <1 | 60.4% (59.0%-61.7%) | 39.6% (38.3%-41.0%) | 81.4% (80.3%-82.4%) | 18.6% (17.6%-19.7%) |
| 1 | 62.0% (54.0%-69.4%) | 38.0% (30.6%-46.0%) | 84.1% (78.0%-88.7%) | 15.9% (11.3%-22.0%) |
| >1 | 65.7% (63.9%-67.4%) | 34.3% (32.6%-36.1%) | 83.7% (82.4%-84.9%) | 16.3% (15.1%-17.6%) |
| **Treated Depression** |  |  |  |  |
| Yes | 63.8% (58.2%-69.1%) | 36.2% (30.9%-41.8%) | 72.6% (68.3%-76.5%) | 27.4% (23.5%-31.7%) |
| No | 61.9% (60.8%-63.0%) | 38.1% (37.0%-39.2%) | 83.0% (82.2%-83.7%) | 17.0% (16.3%-17.8%) |
| **AUDIT Dependence score ^b^** |  |  |  |  |
| 0 | 70.5% (69.1%-71.7%) | 29.5% (28.3%-30.9%) | 86.9% (86.1%-87.7%) | 13.1% (12.3%-13.9%) |
| [1-2] | 57.6% (55.4%-59.9%) | 42.4% (40.1%-44.6%) | 74.4% (72.2%-76.5%) | 25.6% (23.5%-27.8%) |
| ≥3 | 43.0% (40.4%-45.6%) | 57.0% (54.4%-59.6%) | 62.4% (59.1%-65.5%) | 37.6% (34.5%-40.9%) |
| **Binge Drinking** |  |  |  |  |
| Never | 71.8% (70.4%-73.2%) | 28.2% (26.8%-29.6%) | 86.5% (85.7%-87.3%) | 13.5% (12.7%-14.3%) |
| At least once | 51.0% (49.4%-52.6%) | 49.0% (47.4%-50.6%) | 67.7% (65.6%-69.7%) | 32.3% (30.3%-34.4%) |
| **Cannabis use** |  |  |  |  |
| Not during the previous 12 months | 64.1% (63.0%-65.2%) | 35.9% (34.8%-37.0%) | 83.5% (82.7%-84.3%) | 16.5% (15.7%-17.3%) |
| At least once during the previous 12 months | 43.7% (40.1%-47.4%) | 56.3% (52.6%-59.9%) | 57.7% (52.7%-62.6%) | 42.3% (37.4%-47.3%) |
| **Smoking Status** |  |  |  |  |
| Non-smoker or occasional smoker | 64.2% (63.1%-65.4%) | 35.8% (34.6%-36.9%) | 83.9% (83.1%-84.7%) | 16.1% (15.3%-16.9%) |
| Smoker | 49.4% (46.2%-52.5%) | 50.6% (47.5%-53.8%) | 71.1% (68.2%-73.7%) | 28.9% (26.3%-31.8%) |
| **Smoking and Vaping** |  |  |  |  |
| No use | 64.6% (63.5%-65.8%) | 35.4% (34.2%-36.5%) | 84.2% (83.4%-85.0%) | 15.8% (15.0%-16.6%) |
| Vape only | 54.4% (47.9%-60.7%) | 45.6% (39.3%-52.1%) | 73.4% (67.2%-78.7%) | 26.6% (21.3%-32.8%) |
| Tobacco only | 50.1% (46.7%-53.5%) | 49.9% (46.5%-53.3%) | 70.8% (67.7%-73.6%) | 29.2% (26.4%-32.3%) |
| Vape and tobacco | 45.8% (38.0%-53.9%) | 54.2% (46.1%-62.0%) | 72.6% (64.4%-79.5%) | 27.4% (20.5%-35.6%) |
| **Self-rated Health ^c^** |  |  |  |  |
| [1-3] | 62.3% (61.1%-63.4%) | 37.7% (36.6%-38.9%) | 82.8% (82.0%-83.6%) | 17.2% (16.4%-18.0%) |
| >3 | 61.1% (58.4%-63.6%) | 38.9% (36.4%-41.6%) | 80.1% (78.1%-82.0%) | 19.9% (18.0%-21.9%) |
| **CVD ^d^** |  |  |  |  |
| Yes | 58.0% (55.4%-60.6%) | 42.0% (39.4%-44.6%) | 83.3% (81.1%-85.4%) | 16.7% (14.6%-18.9%) |
| No | 63.0% (61.9%-64.2%) | 37.0% (35.8%-38.1%) | 82.0% (81.2%-82.9%) | 18.0% (17.1%-18.8%) |
| **Cancer** |  |  |  |  |
| Yes | 64.3% (58.0%-70.1%) | 35.7% (29.9%-42.0%) | 85.2% (80.8%-88.8%) | 14.8% (11.2%-19.2%) |
| No | 61.9% (60.8%-63.0%) | 38.1% (37.0%-39.2%) | 82.1% (81.3%-82.9%) | 17.9% (17.1%-18.7%) |
| ^a^ Effort reward imbalance. (<1: indicating an imbalance in favor of reward, 1: effort reward balance, >1: indicating an imbalance in favor of effort.  ^b^ Alcohol Use Disorder Identification Test, item 3 to 10.  ^c^ Self-rated health from 1= “very good” to 8= “very poor”.  ^d^ Cardiovascular disease. | | | | |

Supplemental Table 5. Sociodemographic characteristics of the population not having at least 2 alcohol-free days per week according to gender in 2019. (weighted percentages and 95% confidence intervals, n=34,470) (The CONSTANCES study, Metropolitan France, 2019)

|  | **At least 2 days per week without alcohol consumption** | | | |
| --- | --- | --- | --- | --- |
|  | **Men** | | **Women** | |
|  | **Not exceeding the recommendations** | **Exceeding the recommendations** | **Not exceeding the recommendations** | **Exceeding the recommendations** |
|  | 8 924 58.4% (57.3%-59.5%) | 6 445 41.6% (40.5%-42.7%) | 14 702 77.4% (76.5%-78.2%) | 4 399 22.6% (21.8%-23.5%) |
| **Age** |  |  |  |  |
| 20-34 | 64.5% (62.0%-66.9%) | 35.5% (33.1%-38.0%) | 79.2% (77.4%-80.9%) | 20.8% (19.1%-22.6%) |
| 35-44 | 63.1% (61.0%-65.3%) | 36.9% (34.7%-39.0%) | 80.0% (78.3%-81.5%) | 20.0% (18.5%-21.7%) |
| 45-54 | 63.1% (60.9%-65.3%) | 36.9% (34.7%-39.1%) | 78.6% (76.8%-80.3%) | 21.4% (19.7%-23.2%) |
| 55-64 | 54.0% (51.5%-56.5%) | 46.0% (43.5%-48.5%) | 75.7% (73.6%-77.6%) | 24.3% (22.4%-26.4%) |
| 65-74 | 44.8% (42.0%-47.5%) | 55.2% (52.5%-58.0%) | 71.1% (68.7%-73.4%) | 28.9% (26.6%-31.3%) |
| **Employment status** |  |  |  |  |
| Employed | 62.2% (60.9%-63.4%) | 37.8% (36.6%-39.1%) | 79.1% (78.1%-80.0%) | 20.9% (20.0%-21.9%) |
| Unemployed | 62.0% (57.9%-65.9%) | 38.0% (34.1%-42.1%) | 77.0% (73.9%-79.9%) | 23.0% (20.1%-26.1%) |
| Retired | 46.9% (44.6%-49.2%) | 53.1% (50.8%-55.4%) | 71.9% (69.9%-73.8%) | 28.1% (26.2%-30.1%) |
| Student | 64.3% (53.6%-73.8%) | 35.7% (26.2%-46.4%) | 81.1% (74.4%-86.4%) | 18.9% (13.6%-25.6%) |
| **Education ^a^** |  |  |  |  |
| Level 0 and Level 1 | 55.6% (49.0%-61.9%) | 44.4% (38.1%-51.0%) | 75.1% (68.4%-80.8%) | 24.9% (19.2%-31.6%) |
| Level 2 | 53.9% (48.9%-58.8%) | 46.1% (41.2%-51.1%) | 75.2% (71.2%-78.8%) | 24.8% (21.2%-28.8%) |
| Level 3 and Level 4 | 58.9% (57.0%-60.8%) | 41.1% (39.2%-43.0%) | 79.7% (78.2%-81.2%) | 20.3% (18.8%-21.8%) |
| Level 5 and Level 6 | 59.0% (57.0%-60.9%) | 41.0% (39.1%-43.0%) | 77.9% (76.5%-79.1%) | 22.1% (20.9%-23.5%) |
| Level 7 and Level 8 | 58.7% (56.7%-60.6%) | 41.3% (39.4%-43.3%) | 73.6% (71.8%-75.3%) | 26.4% (24.7%-28.2%) |
| **Occupational Grade** |  |  |  |  |
| Never worked | 67.1% (58.1%-75.0%) | 32.9% (25.0%-41.9%) | 81.1% (74.5%-86.3%) | 18.9% (13.7%-25.5%) |
| Manual worker or employee | 60.1% (58.3%-61.8%) | 39.9% (38.2%-41.7%) | 79.3% (78.1%-80.5%) | 20.7% (19.5%-21.9%) |
| Intermediate profession | 57.9% (55.6%-60.1%) | 42.1% (39.9%-44.4%) | 76.7% (75.2%-78.2%) | 23.3% (21.8%-24.8%) |
| Executive | 56.1% (54.3%-57.8%) | 43.9% (42.2%-45.7%) | 72.9% (71.2%-74.6%) | 27.1% (25.4%-28.8%) |
| **Household Income** |  |  |  |  |
| <2100 euros | 60.5% (58.1%-62.9%) | 39.5% (37.1%-41.9%) | 78.0% (76.2%-79.7%) | 20.0% (20.3%-23.8%) |
| [2100-2800[ | 59.4% (56.6%-62.1%) | 40.6% (37.9%-43.4%) | 79.2% (77.1%-81.1%) | 20.8% (18.9%-22.9%) |
| [2800-4200[ | 58.2% (56.3%-60.0%) | 41.8% (40.0%-43.7%) | 78.1% (76.6%-79.5%) | 21.9% (20.5%-23.4%) |
| ≥4200 | 55.9% (54.0%-57.8%) | 44.1% (42.2%-46.0%) | 73.9% (72.2%-75.5%) | 26.1% (24.5%-27.8%) |
| **Marital Status** |  |  |  |  |
| Single | 59.9% (58.0%-61.7%) | 40.1% (38.3%-42.0%) | 77.1% (75.8%-78.4%) | 22.9% (21.6%-24.2%) |
| In couple | 57.5% (56.1%-58.8%) | 42.5% (41.2%-43.9%) | 77.5% (76.4%-78.6%) | 22.5% (21.4%-23.6%) |
| **Children** |  |  |  |  |
| Yes | 57.2% (55.8%-58.6%) | 42.8% (41.4%-44.2%) | 77.8% (76.8%-78.8%) | 22.2% (21.2%-23.2%) |
| No | 59.9% (58.2%-61.5%) | 40.1% (38.5%-41.8%) | 76.5% (75.0%-78.0%) | 23.5% (22.0%-25.0%) |
| ^a^ Based on the International Standard Classification of Education. | | | | |

Supplemental Table 6. Clinical characteristics of the population not having at least 2 alcohol-free days per week according to gender in 2019. (weighted percentages and 95% confidence intervals, n=34,470) (The CONSTANCES study, Metropolitan France, 2019)

|  | **At least 2 days per week without alcohol consumption** | | | |
| --- | --- | --- | --- | --- |
|  | **Men** | | **Women** | |
|  | **Not exceeding the recommendations** | **Exceeding the recommendations** | **Not exceeding the recommendations** | **Exceeding the recommendations** |
|  | 8 924 58.4% (57.3%-59.5%) | 6 445 41.6% (40.5%-42.7%) | 14 702 77.4% (76.5%-78.2%) | 4 399 22.6% (21.8%-23.5%) |
| **Pregnant** |  |  |  |  |
| Yes |  |  | 95.6% (92.5%-97.5%) | 4.4% (2.5%-7.5%) |
| No |  |  | 76.9% (76.0%-77.7%) | 23.1% (22.3%-24.0%) |
| **ERI ^a^** |  |  |  |  |
| <1 | 56.6% (55.2%-58.0%) | 43.4% (42.0%-44.8%) | 76.3% (75.2%-77.4%) | 23.7% (22.6%-24.8%) |
| 1 | 50.3% (42.3%-58.3%) | 49.7% (41.7%-57.7%) | 78.0% (71.2%-83.5%) | 22.0% (16.5%-28.8%) |
| >1 | 62.8% (61.1%-64.6%) | 37.2% (35.4%-38.9%) | 79.3% (77.9%-80.5%) | 20.7% (19.5%-22.1%) |
| **Treated Depression** |  |  |  |  |
| Yes | 63.8% (58.1%-69.1%) | 36.2% (30.9%-41.9%) | 71.5% (67.3%-75.4%) | 28.5% (24.6%-32.7%) |
| No | 58.1% (57.0%-59.2%) | 41.9% (40.8%-43.0%) | 77.8% (76.9%-78.6%) | 22.2% (21.4%-23.1%) |
| **AUDIT Dependence score ^b^** |  |  |  |  |
| 0 | 64.0% (62.7%-65.4%) | 36.0% (34.6%-37.3%) | 80.9% (79.9%-81.8%) | 19.1% (18.2%-20.1%) |
| [1-2] | 54.3% (52.0%-56.5%) | 45.7% (43.5%-48.0%) | 71.0% (68.8%-73.1%) | 29.0% (26.9%-31.2%) |
| ≥3 | 47.1% (44.5%-49.8%) | 52.9% (50.2%-55.5%) | 63.7% (60.5%-66.8%) | 36.3% (33.2%-39.5%) |
| **Binge Drinking** |  |  |  |  |
| Never | 64.8% (63.4%-66.2%) | 35.2% (33.8%-36.6%) | 80.7% (79.8%-81.6%) | 19.3% (18.4%-20.2%) |
| At least once | 51.2% (49.6%-52.8%) | 48.8% (47.2%-50.4%) | 66.0% (64.0%-68.0%) | 34.0% (32.0%-36.0%) |
| **Cannabis use** |  |  |  |  |
| Not during the previous 12 months | 60.2% (59.1%-61.3%) | 39.8% (38.7%-40.9%) | 78.2% (77.4%-79.4%) | 21.8% (20.9%-22.6%) |
| At least once during the previous 12 months | 42.6% (39.0%-46.3%) | 57.4% (53.7%-61.0%) | 60.3% (55.4%-65.0%) | 39.7% (35.0%-44.6%) |
| **Smoking Status** |  |  |  |  |
| Non-smoker or occasional smoker | 60.1% (58.9%-61.2%) | 39.9% (38.8%-41.1%) | 78.8% (77.9%-79.6%) | 21.2% (20.4%-22.1%) |
| Smoker | 48.8% (45.7%-52.0%) | 51.2% (48.0%-54.3%) | 68.0% (65.1%-70.7%) | 32.0% (29.3%-34.9%) |
| **Smoking and Vaping** |  |  |  |  |
| No use | 60.4% (59.3%-61.6%) | 39.6% (38.4%-40.7%) | 79.0% (78.1%-79.9%) | 21.0% (20.1%-21.9%) |
| Vape only | 51.3% (44.8%-57.7%) | 48.7% (42.3%-55.2%) | 70.7% (64.4%-76.2%) | 29.3% (23.8%-35.6%) |
| Tobacco only | 48.0% (44.6%-51.5%) | 52.0% (48.5%-55.4%) | 67.3% (64.2%-70.3%) | 32.7% (29.7%-35.8%) |
| Vape and tobacco | 52.7% (44.6%-60.7%) | 47.3% (39.3%-55.4%) | 71.5% (63.1%-78.6%) | 28.5% (21.4%-36.9%) |
| **Self-rated Health ^c^** |  |  |  |  |
| [1-3] | 58.6% (57.4%-59.8%) | 41.4% (40.2%-42.6%) | 77.7% (76.8%-78.6%) | 22.3% (21.4%-23.2%) |
| >3 | 57.6% (55.0%-60.2%) | 42.4% (39.8%-45.0%) | 76.1% (73.9%-78.1%) | 23.9% (21.9%-26.1%) |
| **CVD ^d^** |  |  |  |  |
| Yes | 54.7% (52.0%-57.3%) | 45.3% (42.7%-48.0%) | 77.1% (74.6%-79.4%) | 22.9% (20.6%-25.4%) |
| No | 59.4% (58.2%-60.6%) | 40.6% (39.4%-41.8%) | 77.4% (76.5%-78.3%) | 22.6% (21.7%-23.5%) |
| **Cancer** |  |  |  |  |
| Yes | 58.8% (52.4%-64.9%) | 48.2% (44.8%-51.6%) | 80.0% (75.1%-84.2%) | 20.0% (15.8%-24.9%) |
| No | 58.4% (57.3%-59.5%) | 41.6% (40.5%-42.7%) | 77.3% (76.4%-78.1%) | 22.7% (21.9%-23.6%) |
| ^a^ Effort reward imbalance. (<1: indicating an imbalance in favor of reward, 1: effort reward balance, >1: indicating an imbalance in favor of effort.  ^b^ Alcohol Use Disorder Identification Test, item 3 to 10.  ^c^ Self-rated health from 1= “very good” to 8= “very poor”.  ^d^ Cardiovascular disease. | | | | |

Supplemental Table 7. Sociodemographic characteristics of the population exceeding at least one of the 3 critetion from the low-risk drinking guidelines according to gender in 2019. (weighted percentages and 95% confidence intervals, n=32,820)(The CONSTANCES study, Metropolitan France, 2019)

|  | **Exceeding the recommendations** | | | |
| --- | --- | --- | --- | --- |
|  | **Men** | | **Women** | |
|  | **Not exceeding the recommendations** | **Exceeding the recommendations** | **Not exceeding the recommendations** | **Exceeding the recommendations** |
|  | 5 992 41.0% (39.9%-42.1%) | 8 734 59.0% (57.9%-60.1%) | 11 987 66.9% (66.0%-67.9%) | 6 107 33.1% (32.1%-34.0%) |
| **Age** |  |  |  |  |
| 20-34 | 42.6% (40.0%-45.2%) | 57.4% (54.8%-60.0%) | 63.3% (61.1%-65.5%) | 36.7% (34.5%-38.9%) |
| 35-44 | 42.6% (40.3%-44.8%) | 57.4% (55.2%-59.7%) | 66.7% (64.8%-68.6%) | 33.3% (31.4%-35.2%) |
| 45-54 | 44.4% (42.1%-46.7%) | 55.6% (53.3%-57.9%) | 69.6% (67.7%-71.6%) | 30.4% (28.4%-32.3%) |
| 55-64 | 40.1% (37.6%-42.6%) | 59.9% (57.4%-62.4%) | 69.0% (66.8%-71.1%) | 31.0% (28.9%-33.2%) |
| 65-74 | 34.1% (31.5%-36.9%) | 65.9% (63.1%-68.5%) | 66.8% (64.2%-69.4%) | 33.2% (30.6%-35.8%) |
| **Employment status** |  |  |  |  |
| Employed | 42.2% (40.9%-43.5%) | 57.8% (56.5%-59.1%) | 66.6% (65.4%-67.7%) | 33.4% (32.3%-34.6%) |
| Unemployed | 47.9% (43.6%-52.2%) | 52.1% (47.8%-56.4%) | 69.8% (66.4%-73.0%) | 30.2% (27.0%-33.6%) |
| Retired | 34.4% (32.1%-36.7%) | 65.6% (63.3%-67.9%) | 67.1% (64.9%-69.2%) | 32.9% (30.8%-35.1%) |
| Student | 53.4% (42.2%-64.2%) | 46.6% (35.8%-57.8%) | 62.2% (53.9%-69.9%) | 37.8% (30.1%-46.1%) |
| **Education ^a^** |  |  |  |  |
| Level 0 and Level 1 | 48.5% (41.4%-55.7%) | 51.5% (44.3%-58.6%) | 82.4% (75.8%-87.6%) | 17.6% (12.4%-24.2%) |
| Level 2 | 39.1% (34.2%-44.2%) | 60.9% (55.8%-65.8%) | 69.7% (65.1%-74.0%) | 30.3% (26.0%-34.9%) |
| Level 3 and Level 4 | 41.8% (39.9%-43.7%) | 58.2% (56.3%-60.1%) | 71.3% (69.5%-73.0%) | 28.7% (27.0%-30.5%) |
| Level 5 and Level 6 | 40.5% (38.5%-42.5%) | 59.5% (57.5%-61.5%) | 66.1% (64.6%-67.6%) | 33.9% (32.4%-35.4%) |
| Level 7 and Level 8 | 39.9% (37.9%-41.9%) | 60.1% (58.1%-62.1%) | 59.1% (57.1%-61.0%) | 40.9% (39.0%-42.9%) |
| **Occupational Grade** |  |  |  |  |
| Never worked | 56.5% (46.8%-65.7%) | 43.5% (34.3%-53.2%) | 69.9% (62.1%-76.6%) | 30.1% (23.4%-37.9%) |
| Manual worker or employee | 42.9% (41.0%-44.7%) | 57.1% (55.3%-59.0%) | 70.8% (69.3%-72.2%) | 29.2% (27.8%-30.7%) |
| Intermediate profession | 41.0% (38.8%-43.3%) | 59.0% (56.7%-61.2%) | 66.7% (65.0%-68.4%) | 33.3% (31.6%-35.0%) |
| Executive | 37.8% (36.1%-39.5%) | 62.2% (60.5%-63.9%) | 58.1% (56.2%-60.0%) | 41.9% (40.0%-43.8%) |
| **Household Income** |  |  |  |  |
| <2100 euros | 47.2% (44.7%-49.8%) | 52.8% (50.2%-55.3%) | 69.3% (67.3%-71.3%) | 30.7% (28.7%-32.7%) |
| [2100-2800[ | 40.0% (37.2%-42.9%) | 60.0% (57.1%-62.8%) | 70.9% (68.6%-73.1%) | 29.1% (26.9%-31.4%) |
| [2800-4200[ | 40.0% (38.2%-41.9%) | 60.0% (58.1%-61.8%) | 66.7% (65.0%-68.3%) | 33.3% (31.7%-35.0%) |
| ≥4200 | 36.9% (35.0%-38.8%) | 63.1% (61.2%-65.0%) | 61.0% (59.2%-62.8%) | 39.0% (37.2%-40.8%) |
| **Marital Status** |  |  |  |  |
| Single | 42.9% (41.0%-44.8%) | 57.1% (55.2%-59.0%) | 65.2% (63.7%-66.8%) | 34.8% (33.2%-36.3%) |
| In a relationship | 39.8% (38.4%-41.1%) | 60.2% (58.9%-61.6%) | 68.2% (67.0%-69.4%) | 31.8% (30.6%-33.0%) |
| **Children** |  |  |  |  |
| Yes | 40.1% (38.7%-41.6%) | 59.9% (58.4%-61.3%) | 68.7% (67.5%-69.8%) | 31.3% (30.2%-32.5%) |
| No | 42.0% (40.3%-43.8%) | 58.0% (56.2%-59.7%) | 63.7% (61.9%-65.4%) | 36.3% (34.6%-38.1%) |
| ^a^ Based on the International Standard Classification of Education. | | | | |

Supplemental Table 8. Clinical characteristics of the population exceeding at least one of the 3 critetion from the low-risk drinking guidelines according to gender in 2019. (weighted percentages and 95% confidence intervals, n=32,820) (The CONSTANCES study, Metropolitan France, 2019)

|  | **Exceeding the recommendations** | | | |
| --- | --- | --- | --- | --- |
|  | **Men** | | **Women** | |
|  | **Not exceeding the recommendations** | **Exceeding the recommendations** | **Not exceeding the recommendations** | **Exceeding the recommendations** |
|  | 5 992 41.0% (39.9%-42.1%) | 8 734 59.0% (57.9%-60.1%) | 11 987 66.9% (66.0%-67.9%) | 6 107 33.1% (32.1%-34.0%) |
| **Pregnant** |  |  |  |  |
| Yes |  |  | 94.6% (90.6%-97.0%) | 5.4% (3.0%-9.4%) |
| No |  |  | 66.2% (65.2%-67.2%) | 33.8% (32.8%-34.8%) |
| **ERI ^a^** |  |  |  |  |
| <1 | 40.1% (38.7%-41.5%) | 59.9% (58.5%-61.3%) | 67.0% (65.8%-68.3%) | 33.0% (31.7%-34.2%) |
| 1 | 30.6% (23.9%-38.3%) | 69.4% (61.7%-76.1%) | 62.1% (54.5%-69.1%) | 37.9% (30.9%-45.5%) |
| >1 | 43.4% (41.6%-45.3%) | 56.6% (54.7%-58.4%) | 66.9% (65.4%-68.4%) | 33.1% (31.6%-34.6%) |
| **Treated Depression** |  |  |  |  |
| Yes | 50.5% (44.6%-56.3%) | 49.5% (43.7%-55.4%) | 63.4% (58.9%-67.6%) | 36.6% (32.4%-41.1%) |
| No | 40.5% (39.4%-41.6%) | 59.5% (58.4%-60.6%) | 67.2% (66.2%-68.1%) | 32.8% (31.9%-33.8%) |
| **AUDIT Dependence score ^b^** |  |  |  |  |
| 0 | 48.9% (47.4%-50.3%) | 51.1% (49.7%-52.6%) | 72.7% (71.7%-73.8%) | 27.3% (26.2%-28.3%) |
| [1-2] | 34.6% (32.3%-36.9%) | 65.4% (63.1%-67.7%) | 54.9% (52.4%-57.3%) | 45.1% (42.7%-47.6%) |
| ≥3 | 25.5% (23.2%-28.0%) | 74.5% (72.0%-76.8%) | 46.2% (42.8%-49.7%) | 53.8% (50.3%-57.2%) |
| **Binge Drinking** |  |  |  |  |
| Never | 53.7% (52.2%-55.2%) | 46.3% (44.8%-47.8%) | 73.9% (72.9%-74.9%) | 26.1% (25.1%-27.1%) |
| At least once | 27.1% (25.6%-28.6%) | 72.9% (71.4%-74.4%) | 43.9% (41.8%-46.1%) | 56.1% (53.9%-58.2%) |
| **Cannabis use** |  |  |  |  |
| Not during the previous 12 months | 43.0% (41.9%-44.2%) | 57.0% (55.8%-58.1%) | 68.8% (67.8%-69.7%) | 31.2% (30.3%-32.2%) |
| At least once during the previous 12 months | 22.8% (19.7%-26.1%) | 77.2% (73.9%-80.3%) | 32.8% (28.1%-37.8%) | 67.2% (62.2%-71.9%) |
| **Smoking Status** |  |  |  |  |
| Non-smoker or occasional smoker | 42.9% (41.7%-44.0%) | 57.1% (56.0%-58.3%) | 69.4% (68.4%-70.4%) | 30.6% (29.6%-31.6%) |
| Smoker | 30.1% (27.2%-33.2%) | 69.9% (66.8%-72.8%) | 50.4% (47.2%-53.5%) | 49.6% (46.5%-52.8%) |
| **Smoking and Vaping** |  |  |  |  |
| No use | 43.2% (42.0%-44.5%) | 56.8% (55.5%-58.0%) | 69.6% (68.6%-70.6%) | 30.4% (29.4%-31.4%) |
| Vape only | 32.8% (27.0%-39.3%) | 67.2% (60.7%-73.0%) | 62.0% (55.5%-68.0%) | 38.0% (32.0%-44.5%) |
| Tobacco only | 30.7% (27.5%-34.1%) | 69.3% (65.9%-72.5%) | 48.9% (45.6%-52.2%) | 51.1% (47.8%-54.4%) |
| Vape and tobacco | 27.2% (20.4%-35.2%) | 72.8% (64.8%-79.6%) | 59.6% (50.6%-67.9%) | 40.4% (32.1%-49.4%) |
| **Self-rated Health ^c^** |  |  |  |  |
| [1-3] | 39.9% (38.7%-41.1%) | 60.1% (58.9%-61.3%) | 65.7% (64.6%-66.7%) | 34.3% (33.3%-35.4%) |
| >3 | 45.4% (42.7%-48.2%) | 54.6% (51.8%-57.3%) | 71.8% (69.5%-74.0%) | 28.2% (26.0%-30.5%) |
| **CVD ^d^** |  |  |  |  |
| Yes | 40.9% (38.3%-43.7%) | 59.1% (56.3%-61.7%) | 72.6% (70.0%-75.1%) | 27.4% (24.9%-30.0%) |
| No | 41.0% (39.8%-42.2%) | 59.0% (57.8%-60.2%) | 66.0% (64.9%-67.0%) | 34.0% (33.0%-35.1%) |
| **Cancer** |  |  |  |  |
| Yes | 48.2% (41.5%-55.1%) | 51.8% (44.9%-58.5%) | 77.8% (72.6%-82.3%) | 22.2% (17.7%-27.4%) |
| No | 40.7% (39.6%-41.8%) | 59.3% (58.2%-60.4%) | 66.6% (65.6%-67.5%) | 33.4% (32.5%-34.4%) |
| ^a^ Effort reward imbalance. (<1: indicating an imbalance in favor of reward, 1: effort reward balance, >1: indicating an imbalance in favor of effort.  ^b^ Alcohol Use Disorder Identification Test, item 3 to 10.  ^c^ Self-rated health from 1= “very good” to 8= “very poor”.  ^d^ Cardiovascular disease. | | | | |
